# Vertebral Osteomyelitis Secondary to Pneumococcal Infection

**DOI:** 10.1155/2018/4878423

**Published:** 2018-10-08

**Authors:** Bindu Gandrapu, Preeyanka Sundar, Paula Aucoin

**Affiliations:** ^1^Internal Medicine, Berkshire Medical Center, Pittsfield, MA, USA; ^2^Division Director of Infectious Diseases, Berkshire Medical Center, Pittsfield, MA, USA

## Abstract

Vertebral osteomyelitis secondary to pneumococcal infection is an uncommon condition caused by *Streptococcus pneumoniae*. Fever, back pain, and raised ESR are common features in the clinical setting. We report a 62-year-old female patient who presented with an unusual presentation. Later on, vertebral osteomyelitis secondary to pneumococcal infection was confirmed at T8, 9 by CT scan, MRI, and cytology. The patient was treated successfully with high-dose ceftriaxone.

## 1. Introduction

Vertebral osteomyelitis secondary to pneumococcal infection is an uncommon condition caused by *Streptococcus pneumoniae* [1]. The patients often present with neck or back pain [2]. Studies have reported that upper respiratory tract infection (URTI), sickle cell anemia, diabetes, heavy alcohol use, and bony trauma may predispose the patients to vertebral osteomyelitis [3]. The diagnosis of osteomyelitis is based on (1) pus aspirated from the bone, (2) positive culture (3) local signs of inflammation, and (4) radiological evidence [4]. For the diagnosis of osteomyelitis, two of the above criteria should be present. In case studies reviewed for this report, vertebral osteomyelitis due to pneumococcus was treated effectively with high-dose ceftriaxone. Herren et al. did a systemic review on the diagnosis and treatment options of vertebral osteomyelitis [5]. This case report presents a lady with hypertension, hyperlipidemia, and vertebral osteomyelitis secondary to pneumococcal infection.

## 2. Case Presentation

A 62-year-old female patient presented after an episode of light-headedness followed by fall, loss of consciousness, and amnesia for several minutes. She gave a history of use of laxatives for the complaints of constipation and lower abdominal discomfort. A week prior, she had an episode of near syncope. She reported gradually progressive back pain of four months for which she left her job at a school cafeteria few months prior. There was no history of fever, cough, night sweats, weight loss, or burning micturation. She smokes half a pack per day for forty years. She denied alcohol intake. On examination in A/E, she was awake, alert, oriented, and afebrile with stable vitals and normal extremities and chest was clear, cardiac exam with a normal sinus rhythm, no murmurs. She had mild paravertebral tenderness, right lower back worse than left.

**Figure 1:**
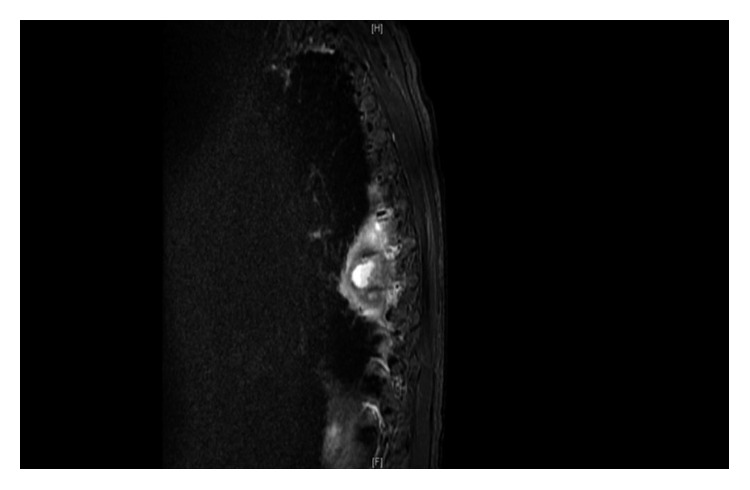
MRI thoracic spine: lateral view reveals discitis osteomyelitis at T8-T9 with an associated 1.6 × 0.8 × 1.5 cm mature rim-enhancing right anterior paravertebal abscess.

**Figure 2:**
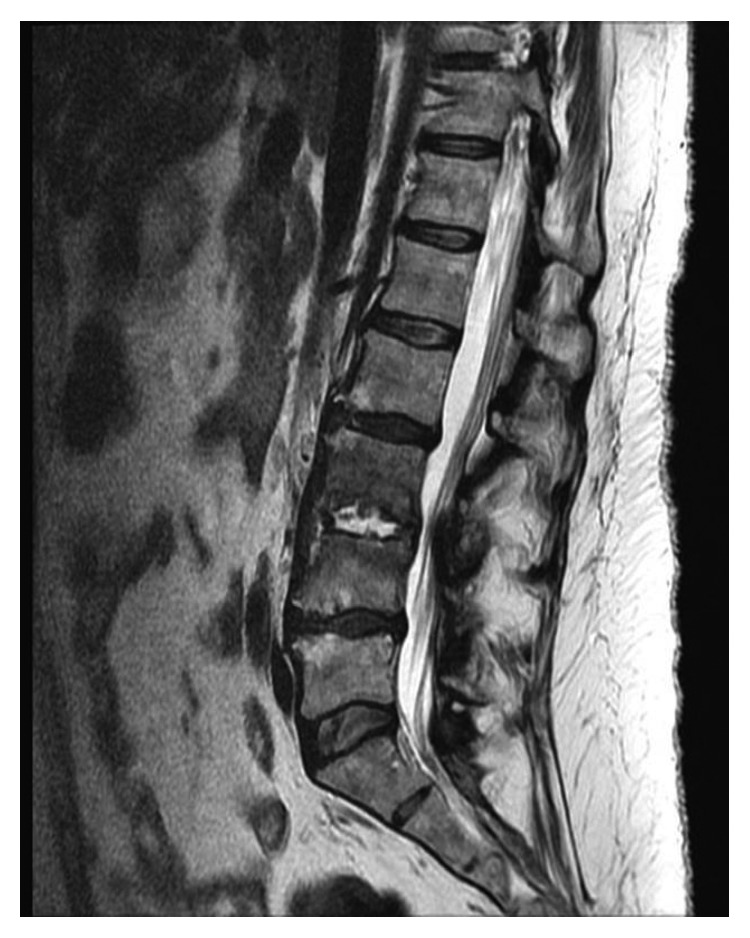
MRI thoracic spine: lateral view reveals discitis osteomyelitis at T8-T9 with an associated 1.6 × 0.8 × 1.5 cm mature rim-enhancing right anterior paravertebal abscess.

CT scan brain plain showed small bilateral SAH in posterior Sylvian fissures and small left IVH. CT scan of thoracic spine indicated sclerosis at T8 and T9 with a large paraspinous mass to the right of the midline at T8 and T9, asymmetric T8-T9 disc space widening. The diagnosis was confirmed on MRI thoracic spine as discitis osteomyelitis at T8-T9 with an associated 1.6 × 0.8 × 1.5 cm mature rim-enhancing right anterior paravertebal abscess (Figures 1 and 2). There was abnormal enhancement and STIR hyperintense signal in the anterior most portion of the T8-T9 disc space with small adjacent endplate erosions along with diffuse marrow edema and enhancement throughout the T8-T9 vertebral bodies. No posteriorly directed epidural abscess in the spinal canal was identified. At T10-T11, a small right central disc protrusion resulting in mild deformity of ventral thecal sac, but no spinal cord impingement, was observed.

**Figure 3:**
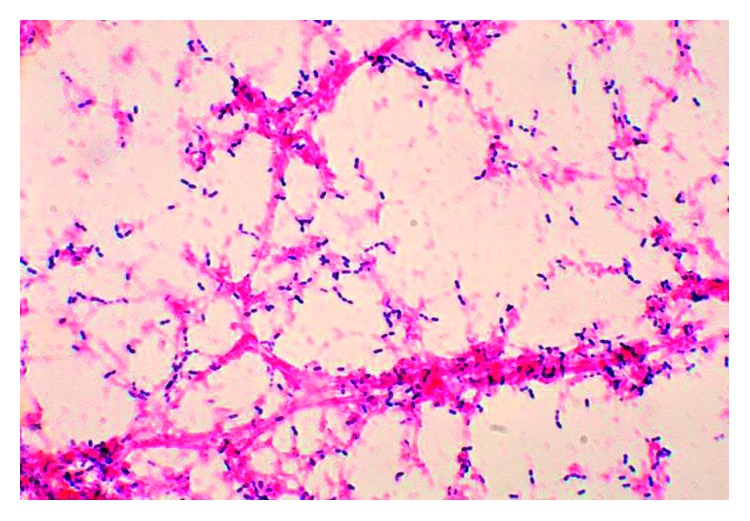
*Streptococcus pneumoniae* Gram stain of blood broth culture.

*S. pneumoniae* was isolated as a direct sample of the spinal tissue biopsy on the primary culture plate, and also she underwent needle aspiration of paravertebral abscess resulting in purulent material from the paraspinous soft tissue mass that cultured streptococcal *pneumoniae* (Figure 3). Lab results showed TLC: 18900/*µ*L, Hb: 13.4 g%, Plt: 379000/*µ*L, erythrocyte sedimentation rate (ESR): 78 (normal 0–30), and CRP: 82 (normal 0–5). She had normal serum protein electrophoresis (SPEP), quantitative immunoglobulins, negative blood cultures, and negative transesophageal echocardiogram (TEE). Gallium scan was performed to rule out alternative etiologies for bacteremia, which indicated uptake in the pubic area. MRI pubis symphysis confirmed osteomyelitis and septic arthritis of the pubic symphysis and pubic rami. The rate of penicillin resistance was high with *S. pneumoniae*, and resistance to ceftriaxone is negligible. She was treated with Unasyn as inpatient transitioned to high-dose ceftriaxone (2g IV 12 hourly) for 6 weeks. She subsequently transitioned to Keflex (500 mg four times a day) for 4 weeks. Repeat CT scan showed resolution of the abscess. She had no neurological sequelae, no apparent adverse effects of medication, and significant decrease in pain.

## 3. Discussion

Vertebral osteomyelitis secondary to streptococcal pneumococcus is a rare complication of invasive pneumococcal infection. The only literature available is case reports and small case series. Fever, back pain, and raised ESR are common features in the clinical setting [3]. Radiological findings include loss of disc height, cortical bone erosion, and epidural abscess formation [6]. In our case, discitis osteomyelitis at T8-T9 with right anterior paravertebal abscess was identified without invasion of the spinal canal.

Suzuki et al. [1] retrospectively identified 14 cases (with median age of 69 years) out of 208 cases with invasive pneumococcal disease (IPD) presented at three teaching hospitals in Japan. They reported 6.4% cases with pneumococcal vertebral osteomyelitis among 208 cases of IPD, affecting mainly lumbar spine (64%) and cervical spine (29%). Reported symptoms include fever (93%) and back/neck pain (86%). Risk factors include smoking (42%), diabetes (36%), heavy alcohol intake (29%), malignancy (14.3%), and immunocompression (14.3%).

In contrast, osteomyelitis was reported at thoracic spine in our case. Osteomyelitis at thoracic spine is very rare entity. Schleiter and Gantz [6] reported one case of osteomyelitis at T4-5, Turner et al. [7] reported 8 cases at T1, T9-10 between 1985 and 1997, Englert et al. [8] reported one case at T8, and Christensen et al. [9] reported one case at T10-11. Hence, our case report is an important addition to the literature on pneumococcal vertebral osteomyelitis affecting thoracic spine.

There are no guidelines available regarding the duration of treatment. Suzuki et al. [1] have reported average duration of antibiotic therapy as 52 days. We offered a high dose of ceftriaxone, which worked well, and the patient's condition improved.

## 4. Conclusion

Vertebral osteomyelitis secondary to streptococcal *pneumoniae* infection is rare, and diagnosis is challenging due to its uncommon presentation.
